# Association between outdoor temperature and bath-related drowning deaths in Japan (1995–2020): modifying factors and the role of prefectural characteristics

**DOI:** 10.1265/ehpm.25-00116

**Published:** 2025-07-18

**Authors:** Yoshiaki Tai, Kenji Obayashi, Yuki Yamagami, Keigo Saeki

**Affiliations:** Department of Epidemiology, Nara Medical University School of Medicine, Nara, Japan

**Keywords:** Bath-related deaths, Outdoor temperature, Accidental drowning, Older population, Housing insulation

## Abstract

**Background:**

Older adults in Japan have the highest drowning mortality rate globally due to frequent bathing practices. Low outdoor temperatures have been linked to bath-related deaths; however, previous studies employed limited statistical models and focused on a single prefecture. Given Japan’s aging population, preventing bath-related deaths is a public health priority. This study aimed to analyze the association between outdoor temperature and bath-related drowning deaths across Japan from 1995 to 2020 (n = 110,938), examining regional variations and identifying contributing prefectural characteristics.

**Methods:**

Daily counts of bath-related drowning deaths per prefecture were matched with daily mean temperature data from the Japan Meteorological Agency. Prefecture-level demographic and environmental data were obtained from Japan’s Official Statistics. We applied a generalized additive mixed model to examine the association between daily mean temperature and bath-related drowning death risk. Meta-regression was used to identify prefecture-level modifiers.

**Results:**

Bath-related drowning death risk peaked at a daily mean temperature of 1.8 °C (relative risk [RR] 9.7, 95% confidence interval [CI]: 9.5–9.9), compared with the lowest risk at 30.3 °C. The association was stronger at mid-range temperatures, particularly among males and individuals aged ≥65 years. Among prefectures, Kagoshima—the southernmost prefecture on Japan’s main islands—had the highest maximum RR at 19.6 (95% CI: 16.2–23.6), while Hokkaido—the northernmost prefecture—had the lowest at 3.8 (95% CI: 3.4–4.3). Prefecture-level factors that strengthened this relationship included a lower prevalence of double-pane windows as a proxy of housing insulation and higher annual mean temperatures with ratio of RR change per one standard deviation increase of 0.76 (95% CI: 0.69–0.83) and 1.27 (95% CI: 1.18–1.37), respectively.

**Conclusions:**

Warmer prefectures in southern regions exhibited greater maximum-to-minimum risk ratios compared to cooler northern prefectures. This paradoxical finding underscores the importance of region-specific interventions to reduce bath-related deaths.

**Supplementary information:**

The online version contains supplementary material available at https://doi.org/10.1265/ehpm.25-00116.

## 1. Background

Low outdoor temperatures have consistently been linked to increased mortality, primarily due to cardiovascular disease, stroke, and respiratory illness [1–3]. Based on a previous study that estimated an attributable fraction of 9.81% [2], the number of excess deaths associated with low temperatures in Japan can be approximated as 134,400 annually, given the total deaths in 2020 (1.37 million) [4]. Additionally, bath-related deaths—sudden fatalities occurring during hot-tub bathing from any cause—exhibit a distinct seasonal peak in winter [5]. In 2020, the estimated increase in bath-related deaths from October to March, compared to April to September, was approximately 13,000 [6], accounting for about 10% of all excess deaths attributable to cold temperatures. These findings highlight the significant contribution of bath-related deaths to the overall increase in mortality during colder months in Japan.

Preventing bath-related deaths based on pathophysiology remains challenging due to the complex and poorly understood underlying mechanisms, as well as the difficulty in distinguishing between endogenous and exogenous causes [6]. Additionally, low autopsy rates in Japan further complicate accurate diagnoses [7]. Although bath-related deaths become more prevalent with age, autopsy rates decline as the age of the deceased increases [8]. Therefore, adopting an environmental approach alongside a pathophysiological one may enhance proactive measures for preventing bath-related deaths.

Previous studies have shown an inverse association between outdoor temperature and the risk of bath-related deaths [9–12]. However, each study was limited to a single prefecture, relatively short observation period, and employed simple linear regression [9, 12] or Poisson regression models [10, 11]. Additionally, the correlation between indoor temperature—which is related to hemodynamic changes during bathing [13]—and outdoor temperature weakens in cold outdoor temperature ranges compared with warmer temperatures [14]. Accordingly, the association between outdoor temperature and the risk of bath-related deaths does not necessarily follow a linear or exponentially increasing trend, nor does it consistently peak at extremely cold temperatures. Moreover, it remains uncertain whether this association is modified by age, sex, and period in a large-scale population, which temperature metrics, including diurnal temperature range or day-to-day fluctuations, best capture it, and whether regional variations exist.

This study offers a novel contribution by leveraging comprehensive death certificate data spanning 26 years across all 47 prefectures in Japan—making it the first study to evaluate regional variability in the temperature–bath-related death association at this scale. In addition, we applied a generalized additive mixed model (GAMM), which allows greater flexibility in modeling non-linear relationships compared to methods used in previous studies. Thus, we aimed to (1) assess whether the strength of this association varies across temperature ranges and by age, sex, and period (2) identify the most explanatory temperature metric for bath-related death risk and (3) examine regional variations in the temperature–bath-related death association. These findings may be used to inform targeted preventive strategies tailored to specific temperature ranges, demographic groups, and regional contexts to reduce bath-related mortality.

## 2. Methods

### 2.1 Daily number of deaths from accidental drowning and submersion in a bathtub

We collected death certificate data on accidental drowning and submersion while in a bathtub, classified under the International Classification of Diseases, 10th Revision (ICD-10) code W65. This code was selected as it represents the leading cause of bath-related deaths, accounting for approximately 80% of all cases [5], and is the only available code for identifying such deaths in death certificates. However, we could not include deaths that occurred during bathing but were attributed to other diagnoses, such as cardiovascular disease or stroke, since death certificates do not indicate whether these endogenous causes occurred in a bathroom. Data on all death certificates listing ICD-10 code W65 as the cause of death were provided by the Japanese Ministry of Health, Labour and Welfare in accordance with Article 33 of the Statistics Act of Japan. For analysis, we included cases from 1995 onward, as ICD-9 coding was used until 1994. In addition to the ICD-10 code, we obtained information on the decedents’ age, sex, date of death, and residential prefecture. We calculated the daily number of W65-coded deaths by year from 1995 to 2020 for each prefecture.

### 2.2 Daily outdoor temperatures

Daily outdoor temperatures, including the mean, maximum, and minimum values, were obtained from the Japan Meteorological Agency website [15]. The diurnal temperature range was calculated as the difference between the maximum and minimum temperatures, whereas the day-to-day mean temperature change was calculated as the daily mean temperature on days with W65-coded deaths minus the daily mean temperature on the previous day, allowing for both positive and negative values. We used the temperature of the most populated city in each prefecture as a representative value because the average daily number of W65-coded deaths per municipality was too low for statistical modeling—approximately 0.013 cases per district—based on 110,938 total cases divided by 845 districts, 365.25 days, and 26 years (1995–2020).

### 2.3 Population of each prefecture in each year from 1995 to 2020

Yearly population data for each prefecture from 1995 to 2020 were obtained from the Japan Census, which is conducted every 5 years, with estimates for intervening years. These population figures, categorized by prefecture and year, were used as offset variables in the GAMM with a quasi-Poisson distribution.

### 2.4 Prefecture-specific factors

Annual data on demographic, socioeconomic, and housing environmental factors, along with support and care certification status in each prefecture, were obtained from the Portal Site of Official Statistics of Japan (e-Stat) [16]. Demographic factors included the proportion of females, the proportion of individuals aged 15–64 and ≥65 years, and the percentage of households with individuals aged ≥65 years living alone. Socioeconomic factors included the annual economic power index, which reflects the financial capacity of local governments and the proportion of people who graduated from college or university. Housing environment factors included the proportion of detached houses, housing with accessible bathtubs for older adults, and housing with multi-pane glass windows, which serve as an indicator of heat insulation capacity. Additionally, the proportion of individuals aged ≥65 years certified for support and long-term care was used as a proxy for frailty and physical disability.

Of these factors, annual values of the economic power index were available only from 1995 to 2017, as the index was discontinued due to major tax reforms. This index was used because no other unified measure covered the entire study period while reflecting the financial capacity of local governments. Similarly, data on the annual proportion of individuals certified for support and long-term care were available only from 2000–2020, as the long-term care insurance system was introduced in 2000, and no classification framework for support and long-term care existed before then. Additionally, data on the proportion of detached houses and housing with accessible bathtubs for older adults were collected every 5 years from 1998 to 2018, as they were derived from the Housing and Land Survey, conducted every 5 years by the Statistics Bureau of the Ministry of Internal Affairs and Communications [17]. Likewise, data on the proportion of housing with multi-pane glass windows were available only for 2008, 2013, and 2018, as data collection began in 2008 as part of the same survey.

### 2.5 Statistical analysis

Descriptive statistics were presented as means (standard deviations [SDs]) for normally distributed continuous variables, medians (interquartile ranges) for non-normally distributed continuous variables, and counts (%) for categorical variables. Trends in the association between the daily prefecture-level incidence of W65-coded deaths (categorized into four groups) and temperature variables were analyzed using linear regression. Temperature variables included daily mean, maximum, and minimum temperatures, as well as the diurnal temperature range and day-to-day mean temperature change. We applied a GAMM with a quasi-Poisson distribution to assess the association between these variables and the daily number of W65-coded deaths, incorporating the total population of each prefecture per year as an offset variable and a random intercept for each prefecture. As daily population data were unavailable, we assumed a constant population throughout the year. Smoothing parameters and fixed effects were estimated using restricted maximum likelihood. The effective degrees of freedom were calculated to assess the smooth term complexity, while marginal and conditional R^2^ values were computed to evaluate the variance explained by fixed effects alone and in combination with random effects.

The estimated risks of W65-coded deaths were converted to relative risks (RRs), using the lowest risk within the 1^st^–99^th^ percentile of each temperature variable range as the reference. RRs for the association between daily mean temperature and W65-coded death risk were estimated for the total population and the subgroups stratified by sex (male and female), age (15–64, 65–79, and ≥80 years), and three time periods (1995–2003, 2004–2012, and 2013–2020). Given the relatively small annual number of W65-coded deaths per prefecture, we estimated the risk and corresponding RRs using a generalized additive model (GAM) with a quasi-Poisson distribution. Estimated RRs were generated for the entire study period to map peak RR across Japan. Peak RR maps were generated for subgroups stratified by sex (male and female), age group (15–64, 65–79, and ≥80 years), and time period (1995–2003, 2004–2012, and 2013–2020). Additionally, estimated RRs were separated for each of the three time periods to conduct the meta-regression analysis described below.

For each prefecture and time period (1995–2003, 2004–2012, and 2013–2020), we calculated the ratio of the maximum to minimum risk of W65-coded deaths estimated using GAM. This ratio, referred to as the peak RR, served as a single summary measure representing the characteristics of each prefecture. We then applied a mixed-effects meta-regression model using these peak RRs, defined as follows for i = 1, 2, …, 47 (prefectures) and j = 1, 2, 3 (time periods):

$$\log({\mathrm{RR}}_{ij})=\beta_{0}+\beta_{1}(x_{ij}-{\bar{x}}_{i})+\beta_{2}{\bar{x}}_{i}+u_{i}+\varepsilon_{ij}$$

where *RR_ij_* is the within-prefecture-level peak RR representing repeated observations within a prefecture for prefecture *i* during period *j*. *x_ij_* is the within-prefecture level explanatory variable for prefecture *i* during period *j*. ${\bar{x}}_{i}$ is the mean of *x_ij_* for prefecture *i* representing the prefecture-level variable. $x_{ij}-{\bar{x}}_{i}$ is the centered within-prefecture-level variable. *u_i_* represents the random effect for prefecture *i*, accounting for the prefecture mean with natural log-transformed RR. *ε_ij_* is the residual error term. Therefore, *β*_1_ represents the association between deviations from the prefecture mean and RR, while *β*_2_ represents the association between the prefecture mean and RR.

All statistical analyses were performed using R software (version 4.3.3) [18]. GAM/GAMM analyses and R^2^ calculations were conducted using the R packages “mgcv” and “MuMIn,” respectively [19, 20]. P-values were calculated using two-sided tests, with statistical significance set at p < 0.05.

## 3. Results

### 3.1 Data selection and prefecture-level annual statistics

After excluding 101, 43, and 8 missing values for daily mean, maximum, and minimum temperatures, respectively, data for 446,207 days remained, representing 99.97% of the total 446,359 days from January 1, 1995, to December 31, 2020, across 47 prefectures. During this period, 110,938 deaths coded as W65 were recorded nationwide. Of these, 90.0% occurred in residential homes, 6.2% in trade and service areas, 2.3% in unspecified locations, and 0.9% in residential institutions.

Table 1 presents prefecture-level annual statistics for the three periods: 1995–2003, 2004–2012, and 2013–2020. Although no formal statistical analysis was conducted, Table 1 suggests potential upward trends in the annual number and risk of W65-coded deaths, proportion of individuals aged ≥65 years, proportion of single-person households aged ≥65 years, proportion of college or university graduates, and proportion of individuals aged ≥65 years certified for support and long-term care over the three periods. In contrast, potential downward trends were observed in the total population and proportion of detached houses.

**Table 1:** Prefecture-Level Characteristics Across Three Time Periods

	**1995–2003**		**2004–2012**		**2013–2020**	
		
	**Mean/Median**	**SD/IQR**	**Mean/** **Median**	**SD/IQR**	**Mean/** **Median**	**SD/IQR**
Accidental drowning and submersion deaths while in a bathtub						
Annual number (cases)	397	249–614	444	307–760	565	341–989
Annual risk (deaths/million)	26.3	12.2	33.2	16.3	46.3	24.6
Proportion						
Female (%)	52.7	4.3	53.0	4.5	51.5	5.0
Aged 15–64 years (%)	14.8	5.4	12.5	6.6	9.0	4.4
Aged ≥65 years (%)	83.2	4.2	87.0	4.1	90.6	3.9
Demographics						
Total population (million)	1.79	1.19–2.76	1.72	1.16–2.76	1.63	1.10–2.72
Proportion of females (%)	51.6	1.0	51.8	1.1	51.8	1.0
Proportion of people aged 15–64 years (%)	66.2	3.0	62.8	2.5	58.1	2.7
Proportion of people aged ≥65 years (%)	18.6	3.2	23.4	3.0	28.9	3.2
Proportion of single-person households aged ≥65 years (%)	6.1	1.9	8.8	2.0	12.0	2.0
Socioeconomic						
Economic power index (thousand)	44.9	19.6	47.2	20.3	49.0	18.6
Proportion of people who graduated college or university (%)	12.2	3.8	14.7	3.9	18.6	4.5
Housing environment (%)						
Proportion of detached houses	67.7	12.1	66.2	12.1	65.2	12.0
Proportion of housing with accessible bathtubs for the elderly	16.7	3.1	19.8	2.9	16.5	2.6
Proportion of housing with multi-pane windows	—	—	19.4	14.7	25.0	14.0
Outdoor environment						
Annual mean outdoor temperature (ºC)	15.4	2.4	15.6	2.3	15.8	2.3
Support and care certification status (%)						
Proportion of those certified for support	2.0	0.8	4.1	1.3	4.9	1.1
Proportion of those certified for long-term care	11.7	1.7	13.0	1.3	13.6	1.2

The mean and standard deviation (SD) were calculated across 47 prefectures based on prefecture-specific average annual statistics for the periods 1995–2003, 2004–2012, and 2013–2020. The median and IQR were calculated across the 47 prefectures for each of these periods.

SD, standard deviation; IQR, interquartile range.

### 3.2 Association between daily mean temperature and bathtub drowning deaths

#### 3.2.1 Overall population

Table 2 presents associations between the temperature variables and the daily prefecture-level incidence of W65-coded deaths. Lower daily mean, maximum, and minimum temperatures, as well as greater day-to-day mean temperature variations, were significantly associated with increased incidence of W65-coded deaths.

**Table 2:** Daily Prefecture-Level Incidence of Bathtub Drowning Deaths and Temperature Variables

	**Daily Prefecture-Level Incidence of Bathtub Drowning Deaths** **(cases per 10 million)**				

	**0**	**>0, ≤2.72**	**>2.72, ≤5.95**	**>5.94**	***P* trend**
**Number of days**	**(n = 367,314)**	**(n = 26,233)**	**(n = 26,339)**	**(n = 26,321)**	
Temperature, °C, mean (SD)					
Daily mean	16.5 (8.6)	13.6 (7.8)	10.6 (7.1)	10.0 (7.2)	<0.001
Daily maximum	21.1 (8.8)	17.7 (8.0)	15.1 (7.5)	14.5 (7.7)	<0.001
Daily minimum	12.7 (8.9)	9.9 (8.0)	6.6 (7.2)	6.0 (7.2)	<0.001
Diurnal range	8.4 (3.3)	7.8 (2.8)	8.5 (3.3)	8.5 (3.4)	0.443
Day-to-day mean change	−0.01 (1.91)	−0.01 (2.03)	0.03 (2.06)	0.05 (2.09)	<0.001

A linear regression model was used to analyze the trends. Diurnal temperature range was calculated as the difference between the maximum and minimum temperatures on days with bathtub drowning deaths. Day-to-day mean temperature change was calculated as the daily mean temperature on days with bathtub drowning deaths minus the daily mean temperature on the previous day, allowing for both positive and negative values.

SD, standard deviation.

**Fig. 1:**
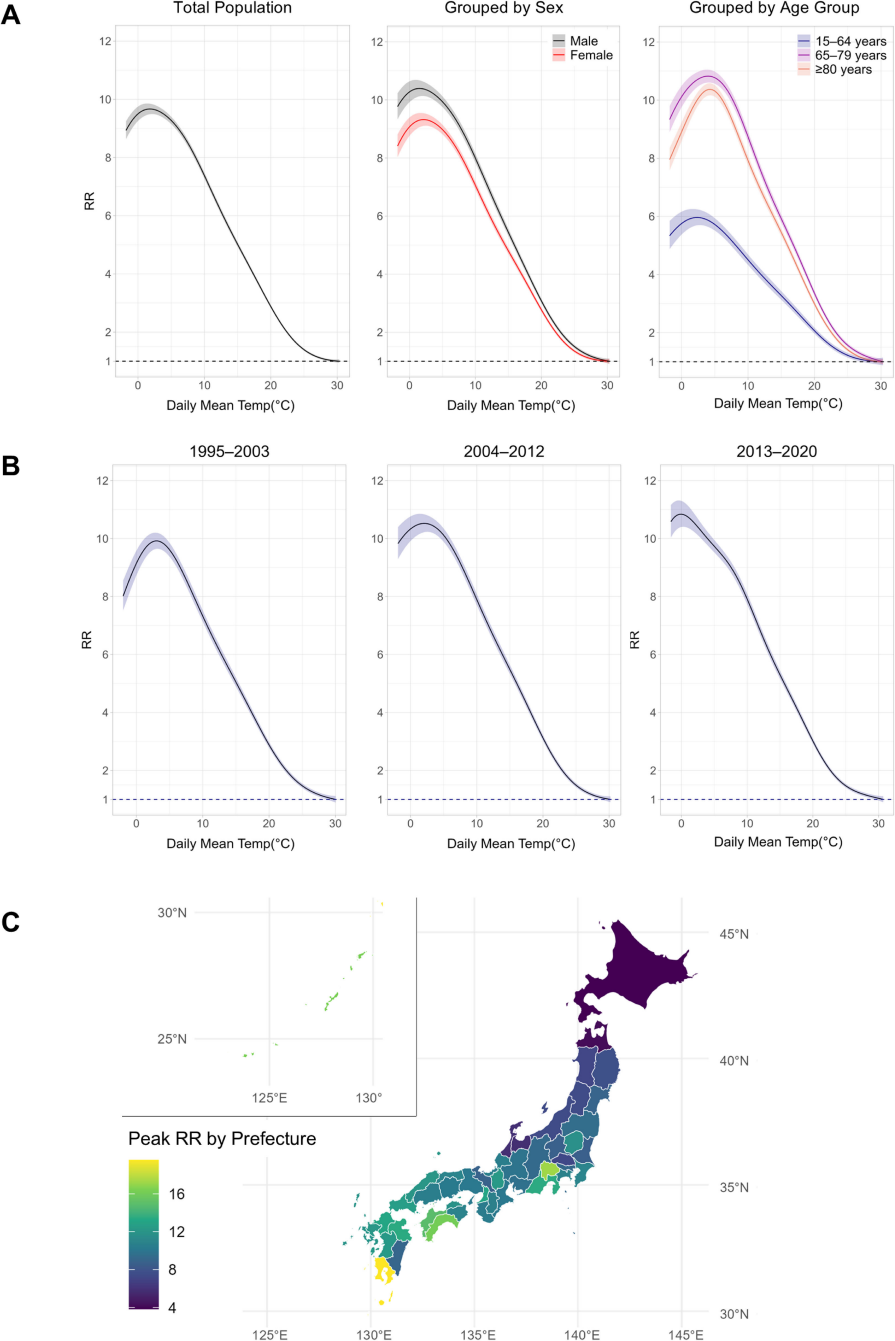
Daily Mean Temperature and Bathtub Drowning Deaths by Sex, Age Group, Time Periods, and Prefectures Panel A: The risk of bathtub drowning deaths was estimated using a generalized additive mixed model with a random intercept for each prefecture. RR values were calculated relative to the minimum predicted risk. RR curves are shown for the total population, stratified by sex and age group. Panel B: RR curves are presented for the periods 1995–2003, 2004–2012, and 2013–2020. Panel C: RR was estimated using a generalized additive model for each prefecture over the entire study period (1995–2020), with peak RR values mapped across Japan. The shaded areas indicate 95% confidence intervals. RR, relative risk.

Figure 1 illustrates the relationship between daily mean temperature and the RR of W65-coded deaths, with the lowest risk set at 1.0, using GAMM with quasi-Poisson regression. The relationship between the daily mean temperature and RR was more pronounced at mid-range temperatures than at higher or lower temperatures. In the total population, the peak RR (ratio of maximum to minimum risk of W65-coded deaths) was 9.7 (95% confidence interval [CI]: 9.5–9.9) at 1.8 °C, with 30.3 °C as the reference (RR = 1.0).

#### 3.2.2 Subgroup analyses by age, sex, and time period

Subgroup analysis showed a stronger association in males and individuals aged 65–79 and ≥80 years than in females and those aged 15–64 years, respectively (Fig. 1A). Over the three study periods, the RR peak shifted slightly toward the upper-left region of the graph, indicating the peak shift toward higher RRs and lower temperatures (Fig. 1B).

#### 3.2.3 Subgroup analyses by prefecture

Southern prefectures generally exhibited higher peak RRs than northern prefectures (Fig. 1C). Similar trends were observed in the subgroup analyses stratified by age, sex, and time period (Figs. S1–S3), except among individuals aged 15–64, for whom some peak RRs exceeded 30. Kagoshima—the southernmost prefecture on Japan’s main islands—had the highest peak RR (19.6, 95% CI: 16.2–23.6), whereas Hokkaido—the northernmost prefecture—had the lowest (3.8, 95% CI: 3.4–4.3). Figures S4–S6 display combined plots of the relationship between the daily mean temperature and RR of W65-coded deaths, along with histograms of W65-coded deaths in each prefecture for the entire study period.

### 3.3 Model performance

Figure S7 displays the relationship between other temperature variables—including daily maximum and minimum temperatures, day-to-day temperature change, and diurnal temperature range—and the RR of W65-coded deaths. Among the temperature variables, the daily mean temperature as a single smooth term had the highest marginal R^2^ (0.216) and conditional R^2^ (0.495) values but also the highest effective degrees of freedom values (Table 3). The marginal R^2^ values for the diurnal temperature range and day-to-day temperature changes were relatively small compared to those for daily mean, maximum, and minimum temperatures.

**Table 3:** Model Performance Metrics for Estimating Bathtub Drowning Risk from Temperature Variables

**Temperature variables**	**EDF**	***P* value**	**Marginal R^2^**	**Conditional R^2^**
Daily mean	8.21	<0.001	0.216	0.495
Daily maximum	8.11	<0.001	0.135	0.434
Daily minimum	8.02	<0.001	0.116	0.378
Diurnal range	5.69	<0.001	0.009	0.038
Day-to-day mean change	6.02	<0.001	0.0003	0.042

A generalized additive mixed model with a random intercept for each prefecture was applied to examine the association between temperature variables and accidental drowning and submersion deaths while in a bathtub. Diurnal temperature range was calculated as the difference between the maximum and minimum temperatures on days with bathtub drowning deaths. Day-to-day mean temperature change was calculated as the daily mean temperature on days with bathtub drowning deaths minus the daily mean temperature on the previous day, allowing for both positive and negative values.

EDF, effective degrees of freedom.

### 3.4 Prefecture-level modifiers of the temperature–drowning association

**Fig. 2:**
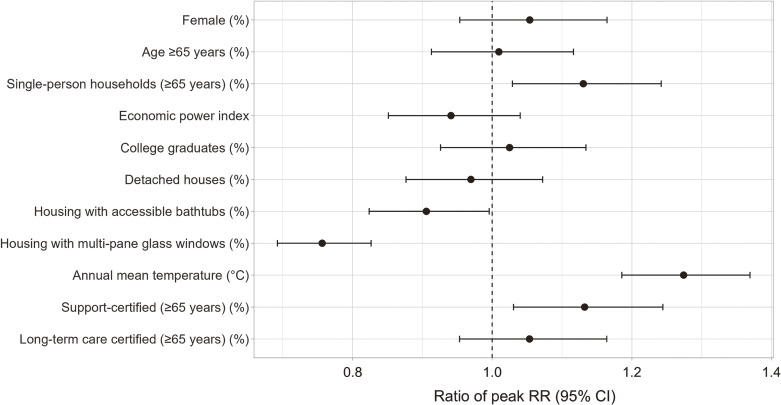
Prefecture-Level Modifiers of the Association Between Daily Mean Temperature and Bathtub Drowning Deaths The risk of bathtub drowning deaths was estimated using a generalized additive model for each prefecture and time period (1995–2003, 2004–2012, and 2013–2020). RR values were then calculated relative to the minimum predicted risk, and the peak RR was identified. The ratio of peak RR was estimated using a meta-regression model, with the natural log-transformed peak RR as the response variable and inter-prefecture changes (one standard deviation) in prefecture-level characteristics as explanatory variables. RR, relative risk.

Figure 2 illustrates the variation in peak RR across different prefecture-level variables. A higher proportion of housing with multi-pane glass windows and accessible bathtubs were significantly associated with a lower peak RR with ratios of change in RR per 1 SD increase of 0.76 (95% CI: 0.69–0.83) and 0.91 (95% CI: 0.82–0.99), respectively. In contrast, a higher proportion of single-person households among those aged ≥65 years, a higher proportion of individuals aged ≥65 years certified for support, and a higher annual mean temperature were significantly associated with a higher peak RR. However, none of the within-prefecture-level variables was significantly associated with the peak RR (Fig. S8).

## 4. Discussion

Using death certificate data from 1995 to 2020 across Japan, we examined the association between outdoor temperature and the risk of accidental drowning and submersion while in a bathtub, labeled as ICD-10 code W65. Our findings indicated that lower daily mean temperatures, particularly mid-range temperatures, were associated with an increased risk of bath-related deaths. Among the temperature variables, the daily mean temperature accounted for the variation in W65-coded deaths most effectively. The inverse association between daily mean temperature and the risk of W65-coded deaths was stronger among males and older individuals. Prefectures with higher mean temperatures and a lower proportion of well-insulated housing had higher peak RRs (maximum-to-minimum RR) for W65-coded deaths. To the best of our knowledge, this is the first study to examine the relationship between outdoor temperature and bath-related deaths across Japan and the factors that influence this association.

Although our findings are consistent with those of previous studies demonstrating an inverse association between outdoor temperature and bath-related deaths, our use of a more flexible modeling approach provides novel insights into this relationship. Earlier investigations assumed linear or log-linear patterns when estimating changes in bath-related deaths as temperatures decreased. One study estimated that a decrease in daily mean temperature from 30 °C to 0 °C corresponded to an RR of bath-related deaths of 32.8 (95% CI, 32.5–33.1) [11]. In contrast, our model showed that the association between daily mean temperature and RR weakens at lower temperatures, with the peak RR reaching only 9.7 as the temperature decreases from 30.3 °C to 1.8 °C. (Fig. 1A). Our results suggest that indoor temperature, rather than outdoor temperature, may play a more significant role in bath-related deaths under cold conditions. Indeed, existing evidence indicates that the correlation between outdoor and indoor temperatures weakens as outdoor temperatures decline [14, 21]. Furthermore, a previous study found that the inverse association between outdoor temperature and the rise in body surface temperature during bathing, which leads to more substantial hemodynamic changes, weakens in warmer indoor environments [13].

The estimated RRs of bath-related deaths for a decrease in daily mean temperature from 30 °C to 0 °C varied across previous studies and are not directly comparable to our findings. Linear regression models used in earlier studies produced negative risk values at 30 °C [9, 12], making RR estimation infeasible. A non-linear model in another study estimated an RR of 8.2 at 0 °C using 30 °C as the reference [10], but insufficient information on model settings limited comparability. Additionally, one study reported an odds ratio of 4.3 for bathing accidents with a 30 °C temperature drop [22], though this was based on ambulance dispatches rather than mortality data. Regarding temperature metrics, our results align with prior studies showing similar explanatory power for daily mean, maximum, and minimum temperatures [9, 12].

Our findings generally align with previous epidemiological and physiological evidence regarding high-risk populations for bath-related deaths. A previous epidemiological study using inquest records found that the incidence of bath-related death was higher among individuals living alone than among those living with family [12]. Similarly, our results showed that prefectures with a higher proportion of single-person households among individuals aged ≥65 years had a higher peak RR. Living alone may increase the risk of fatality due to the absence of witnesses during bathtub drowning incidents. Additionally, the association between outdoor temperature and W65-coded deaths differed significantly between individuals aged 15–64 and those aged ≥65 years (Fig. 1A). Physiological studies suggest that older adults have a reduced ability to regulate blood flow distribution and cardiac output in response to heat stress [23, 24]. This reduced ability to cope with heat exposure may increase the risk of hyperthermia, syncope, and cardiovascular events during bathing, potentially leading to bath-related deaths. Our results indicate that the inverse association between daily mean temperature and the risk of W65-coded deaths was stronger in males than in females. Previous studies have also reported higher bath-related mortality in males [5, 12], which contrasts with epidemiological findings from heatwave investigations, indicating similar mortalities between males and females [25]. This suggests that behavioral factors, such as the higher prevalence of habitual alcohol consumption among males [26], may explain the sex differences in bath-related deaths. Previous epidemiological studies have identified alcohol consumption as a risk factor for bath-related deaths, with some cases attributed to alcohol intoxication as the underlying cause [27, 28]. Furthermore, alcohol-induced impairment of the vasoconstrictive response during orthostatic stress can result in hypotension [29], potentially leading to water aspiration and subsequent drowning [30].

Although the mechanism underlying the higher peak RR of W65-coded deaths in southern and warmer prefectures remains unclear, the indoor temperature may play a key role. An epidemiological study found an association between lower living room temperature and greater heat load during bathing, leading to more pronounced hemodynamic changes [13]. Additionally, a nationwide survey on housing thermal environments reported that Hokkaido, despite its colder climate, had the highest average living room temperature, whereas southern prefectures tended to have lower indoor temperatures despite warmer outdoor conditions [31]. Another survey of thermal conditions in multiple cities found smaller temperature differences between rooms and higher indoor temperatures in Sapporo, the capital of Hokkaido, compared to Akita, Osaka, and Fukuoka—cities located on Japan’s mainland [32]. This suggests that southern regions may have lower insulation levels and/or limited use of heating devices. Consistently, our findings indicate that peak RR was higher in prefectures with a lower proportion of housing with multi-pane windows, an indicator of poor insulation.

Our results showed a shift in the peak of the curve representing the association between the daily mean temperature and the risk of W65-coded deaths at colder temperatures across the three periods (Fig. 1B). However, we did not identify any of the contributing factors. We examined within-prefecture changes in demographic, socioeconomic, and other factors but did not find statistically significant associations with W65-coded deaths (Fig. S5). This may be due to the limited sample size, as the data were grouped into three periods rather than being analyzed annually. We adopted this approach because the number of W65-coded deaths per prefecture was too small in some prefectures (Figs. S1–S3), and calculating peak RR annually for each prefecture would have introduced substantial random error. Additionally, unexamined factors, such as reduced heating due to financial constraints, may have contributed to the observed trend, although relevant data were unavailable.

Our findings have practical implications for preventing bath-related deaths, particularly by raising awareness among high-risk individuals in southern and warmer regions. Residents in these areas may be less aware of the health risks associated with cold exposure. However, our counterintuitive finding of a higher peak RR in warmer regions may prompt a reconsideration of indoor housing environments during winter. Additionally, our results suggest that a lower proportion of housing with accessible bathtubs and a higher proportion of support-certified older adults at the prefectural level may contribute to the risk of bath-related deaths. These two factors may indicate a heightened risk for frail individuals who can bathe independently but face an increased drowning risk without safety devices or supervision.

A key strength of this study is its comprehensive enumeration of W65-coded deaths across Japan from 1995 to 2020. Additionally, the study employed flexible statistical modeling alongside meta-regression to identify factors contributing to inter-prefecture differences in RR increases of W65-coded deaths.

However, this study has some limitations. First, death certificates do not specify bath-related deaths resulting from endogenous conditions, such as cardiovascular events occurring in a bathtub. Consequently, these cases were excluded from our analysis, which may have limited the generalizability of our findings. However, a previous study suggested that drowning and bathtub submersion are the most frequent causes of bath-related deaths [5]. Second, the relatively low annual number of W65-coded deaths per prefecture precluded the calculation of RR estimates at the prefectural level for each year, as discussed earlier. Third, we assessed the temperature–W65-coded deaths association at the population rather than individual level. However, collecting individual-level temperature data at this scale is logistically unfeasible. Fourth, we used daily temperature data from prefectural capitals rather than municipal-level data, which may not have fully reflected temperature variations within a single prefecture. Fifth, the date of death recorded on the death certificates may not always correspond to the actual date of the drowning incident. However, since 99.6% of bath-related deaths occur within 24 hours of bathing [11], any potential mismatch between temperature data and actual exposure is likely minimal. Sixth, annual data on indoor temperature, housing insulation, and energy expenditure for heating were unavailable at the prefectural level. Instead, we used the proportion of houses with double-pane windows as a proxy for heat insulation. Seventh, we assessed effect modification of the temperature–W65-coded deaths association using prefecture-level summary data, which may have introduced ecological fallacy. Eighth, the maximum-to-minimum RR of W65-coded deaths used to assess regional variations was not age-standardized. However, a subgroup analysis among individuals aged 65–79 and ≥80 years revealed a trend similar to that observed in the overall population. Finally, we did not account for lagged effects in the temperature–W65-coded deaths association, based on the assumption that ambient temperature influences bathing behavior and related deaths in the short term.

In conclusion, this study provides novel insights into the association between outdoor temperature and bath-related deaths in Japan. Our findings highlight that lower daily mean temperatures represent a significant risk factor. Moreover, this association is stronger within moderate temperature ranges than at extremely cold temperatures, particularly among males, older individuals—especially those living alone—and residents of southern prefectures. These findings underscore the need for targeted preventive measures to mitigate the impact of environmental temperature on bath-related mortality, particularly among high-risk populations. Future studies should explore the role of indoor temperature in bath-related deaths, particularly in warmer regions, and assess individual-level risk factors.

## Abbreviations

GAM: generalized additive model
GAMM: generalized additive mixed model
ICD: International Classification of Diseases
ICD-10: International Classification of Diseases, 10th Revision
RR: relative risk

## Supplementary information

**Additional file 1:** **Figure S1.** Prefecture-level peak relative risks mapped across Japan by sex. **Figure S2.** Prefecture-level peak relative risks mapped across Japan by age. **Figure S3.** Prefecture-level peak relative risks mapped across Japan by period. **Figure S4.** Association Between Daily Mean Temperature and Bathtub Drowning Deaths in Prefectures (Prefecture No. 1–16). **Figure S5.** Association Between Daily Mean Temperature and Bathtub Drowning Deaths in Prefectures (Prefecture No. 17–32). **Figure S6.** Association Between Daily Mean Temperature and Bathtub Drowning Deaths in Prefectures (Prefecture No. 33–47). **Figure S7.** Association Between Temperature Variables and Bathtub Drowning Deaths. **Figure S8.** Within-Prefecture-Level Possible Modifiers of the Association Between Daily Mean Temperature and Bathtub Drowning Deaths.

## Declarations

### Availability of data and material

Data from individual vital statistics used in this study cannot be shared, as the authors do not have the right to distribute this information. However, the deidentified public data utilized in this study are publicly available and can be accessed directly without request.
