# Supplementary material for: Association between outdoor temperature and bath-related drowning deaths in Japan (1995–2020): modifying factors and the role of prefectural characteristics
Source: Environ Health Prev Med. 2025 Jul 18;30:55. doi: 10.1265/ehpm.25-00116 (PMC12301073; doi:10.1265/ehpm.25-00116)
Supplement: Supplementary file 1 — Additional file 1: Figure S1. Prefecture-level peak relative risks mapped across Japan by sex. Figure S2. Prefecture-level peak relative risks mapped across Japan by age. Figure S3. Prefecture-level peak relative risks mapped across Japan by period. Figure S4. Association Between Daily Mean Temperature and Bathtub Drowning Deaths in Prefectures (Prefecture No. 1–16). Figure S5. Association Between Daily Mean Temperature and Bathtub Drowning Deaths in Prefectures (Prefecture No. 17–32). Figure S6. Association Between Daily Mean Temperature and Bathtub Drowning Deaths in Prefectures (Prefecture No. 33–47). Figure S7. Association Between Temperature Variables and Bathtub Drowning Deaths. Figure S8. Within-Prefecture-Level Possible Modifiers of the Association Between Daily Mean Temperature and Bathtub Drowning Deaths. [file ehpm-30-055-s001.docx]

**Figure S1**. Prefecture-level peak relative risks mapped across Japan by sex


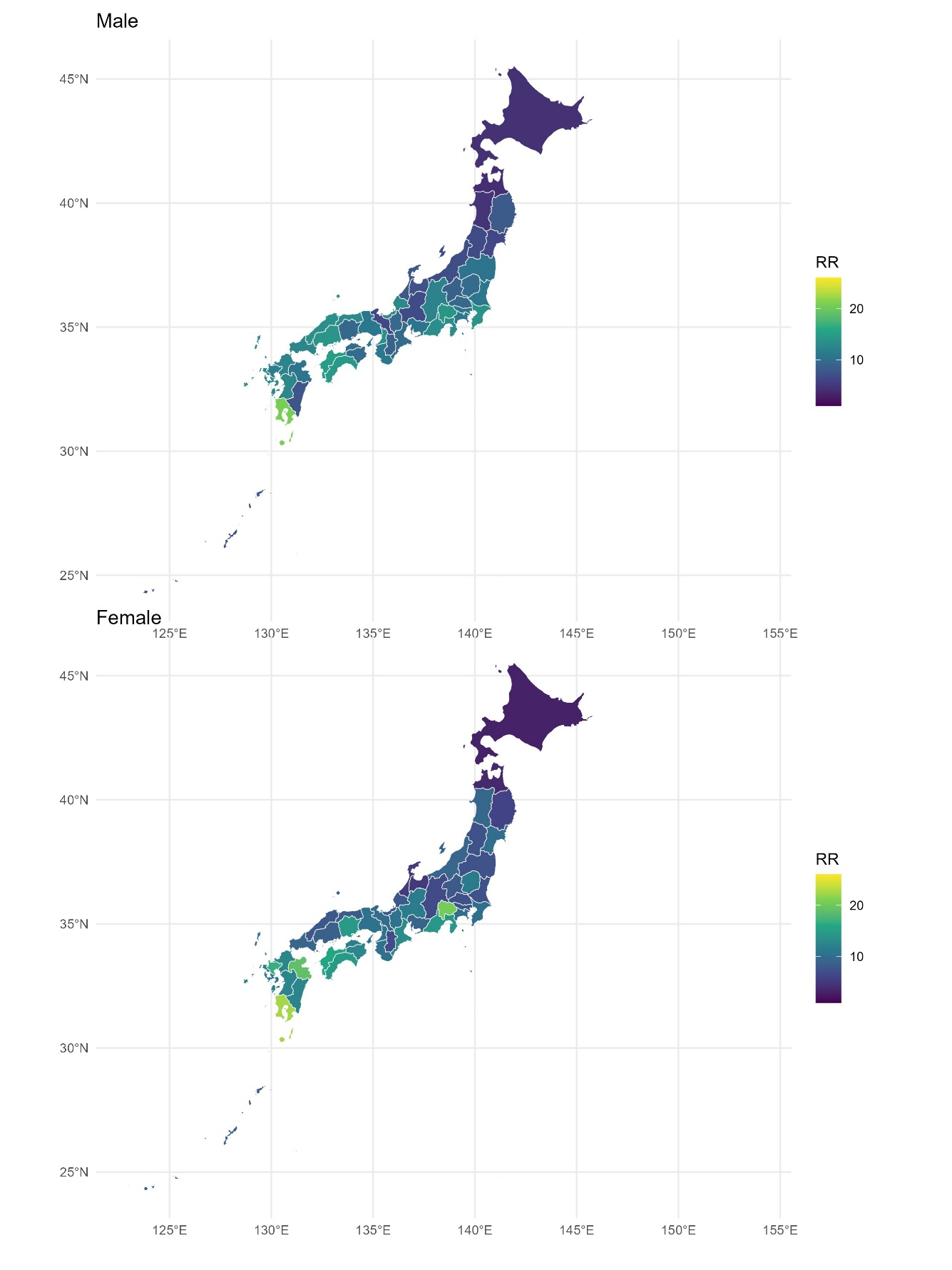


The risk of W65-coded deaths was estimated using a generalized additive model. Peak relative risk was defined as the ratio of maximum to minimum estimated risk

**Figure S2**. Prefecture-level peak relative risks mapped across Japan by age

**
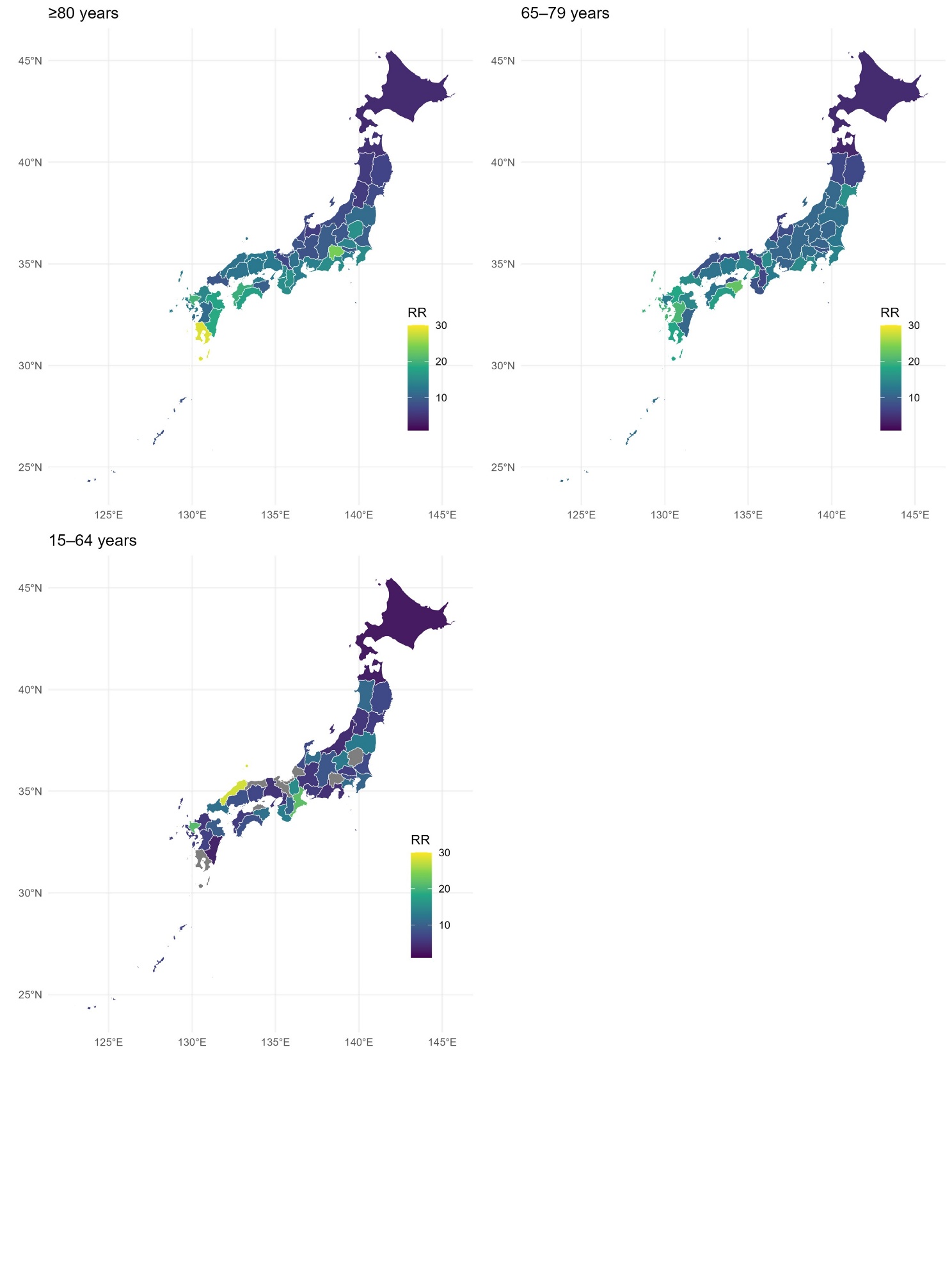
**

The risk of W65-coded deaths was estimated using a generalized additive model. Peak relative risk was defined as the ratio of maximum to minimum estimated risk. Among individuals aged 16–64 years, prefectures with a peak RR above 30 were colored grey to indicate imprecise estimates due to sparse data.

**Figure S3**. Prefecture-level peak relative risks mapped across Japan by period


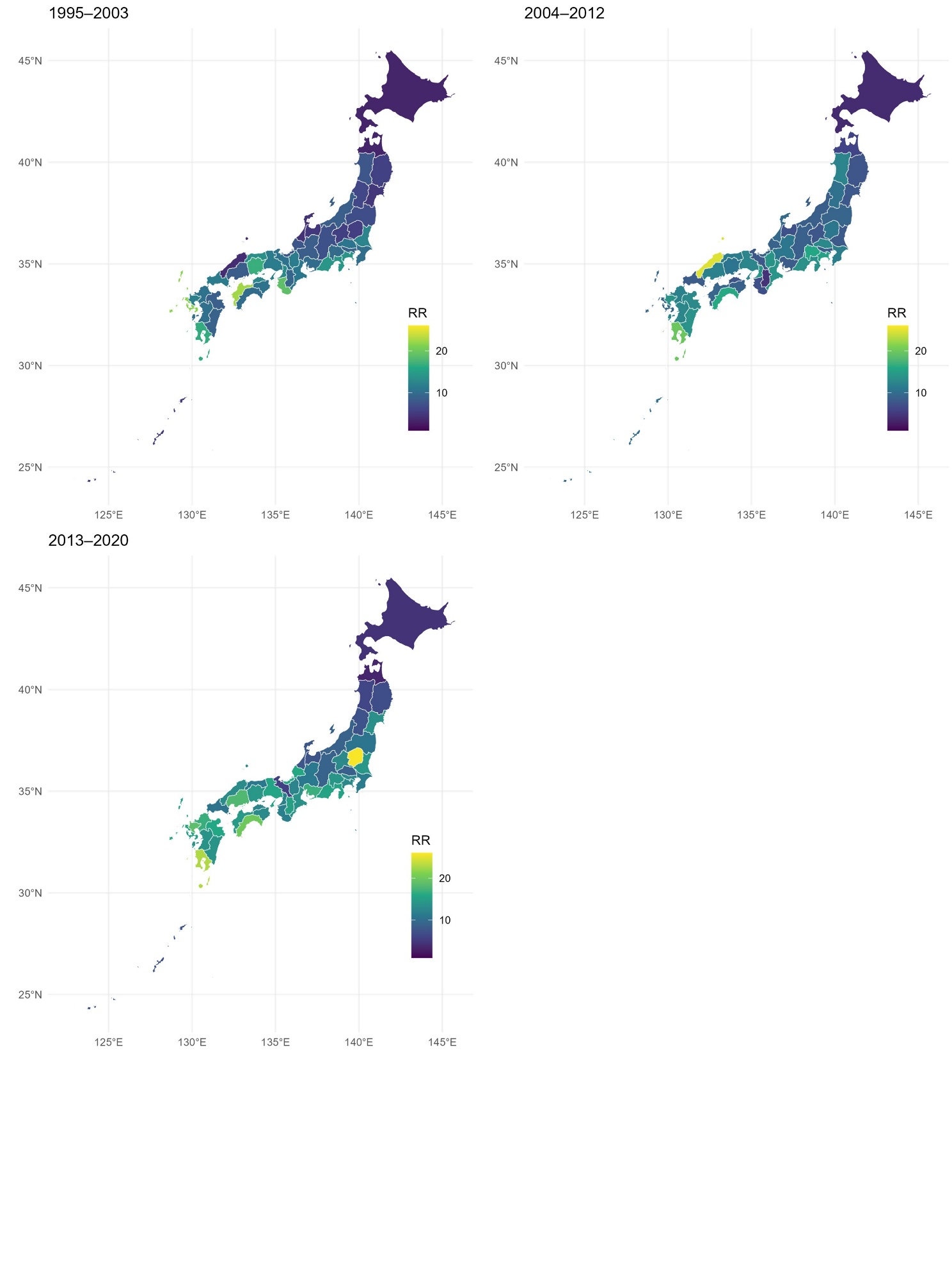


The risk of W65-coded deaths was estimated using a generalized additive model. Peak relative risk was defined as the ratio of maximum to minimum estimated risk.


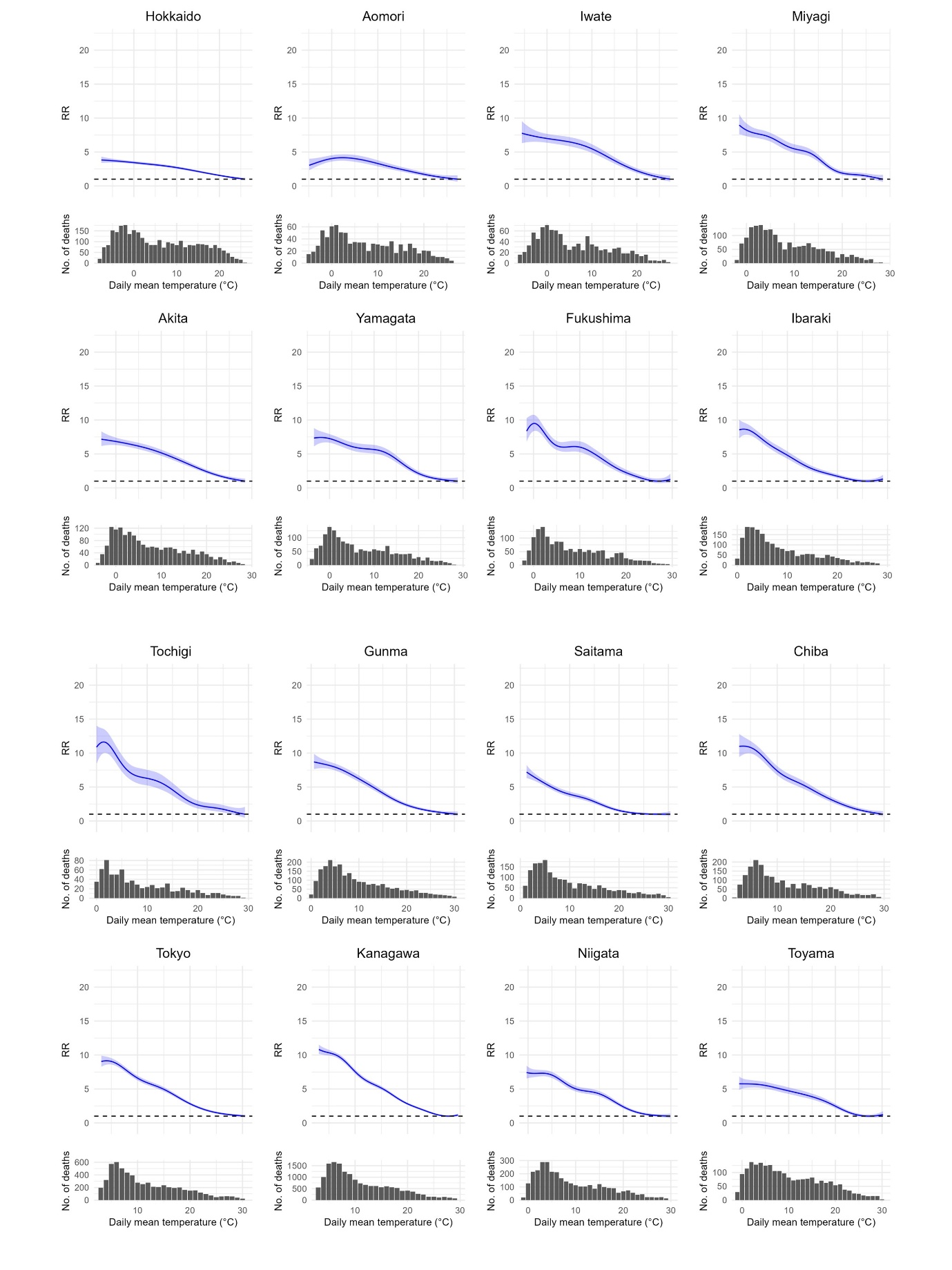
**Figure S4**. Association Between Daily Mean Temperature and Bathtub Drowning Deaths in Prefectures (Prefecture No. 1–16)

The risk of bathtub drowning deaths was estimated using a generalized additive model for each prefecture over the entire study period (1995–2020). RR values were calculated relative to the minimum predicted risk. The shaded areas indicate 95% confidence intervals. RR, relative risk.


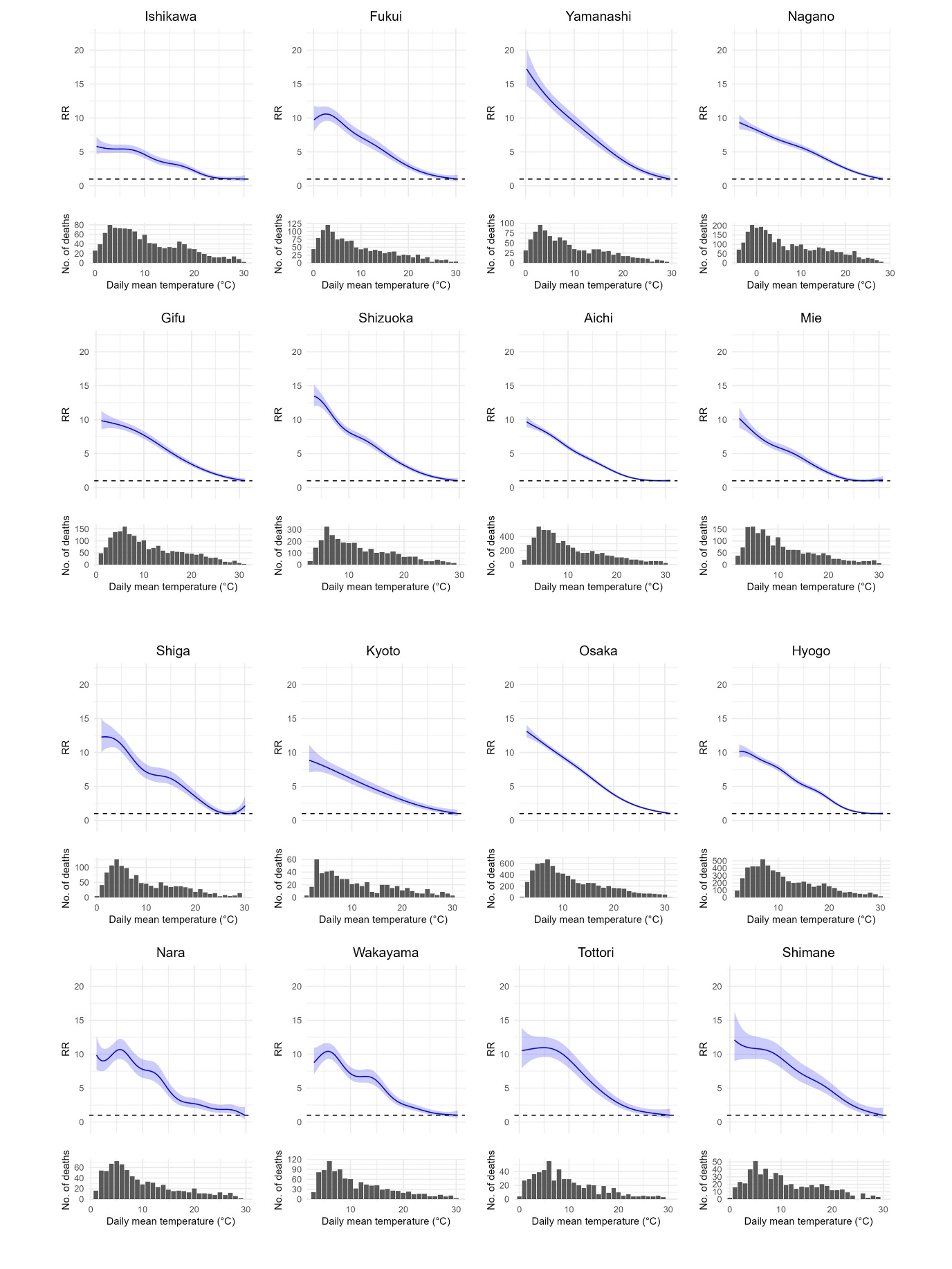
**Figure S5**. Association Between Daily Mean Temperature and Bathtub Drowning Deaths in Prefectures (Prefecture No. 17–32)

The risk of bathtub drowning deaths was estimated using a generalized additive model for each prefecture over the entire study period (1995–2020). RR values were calculated relative to the minimum predicted risk. The shaded areas indicate 95% confidence intervals. RR, relative risk.


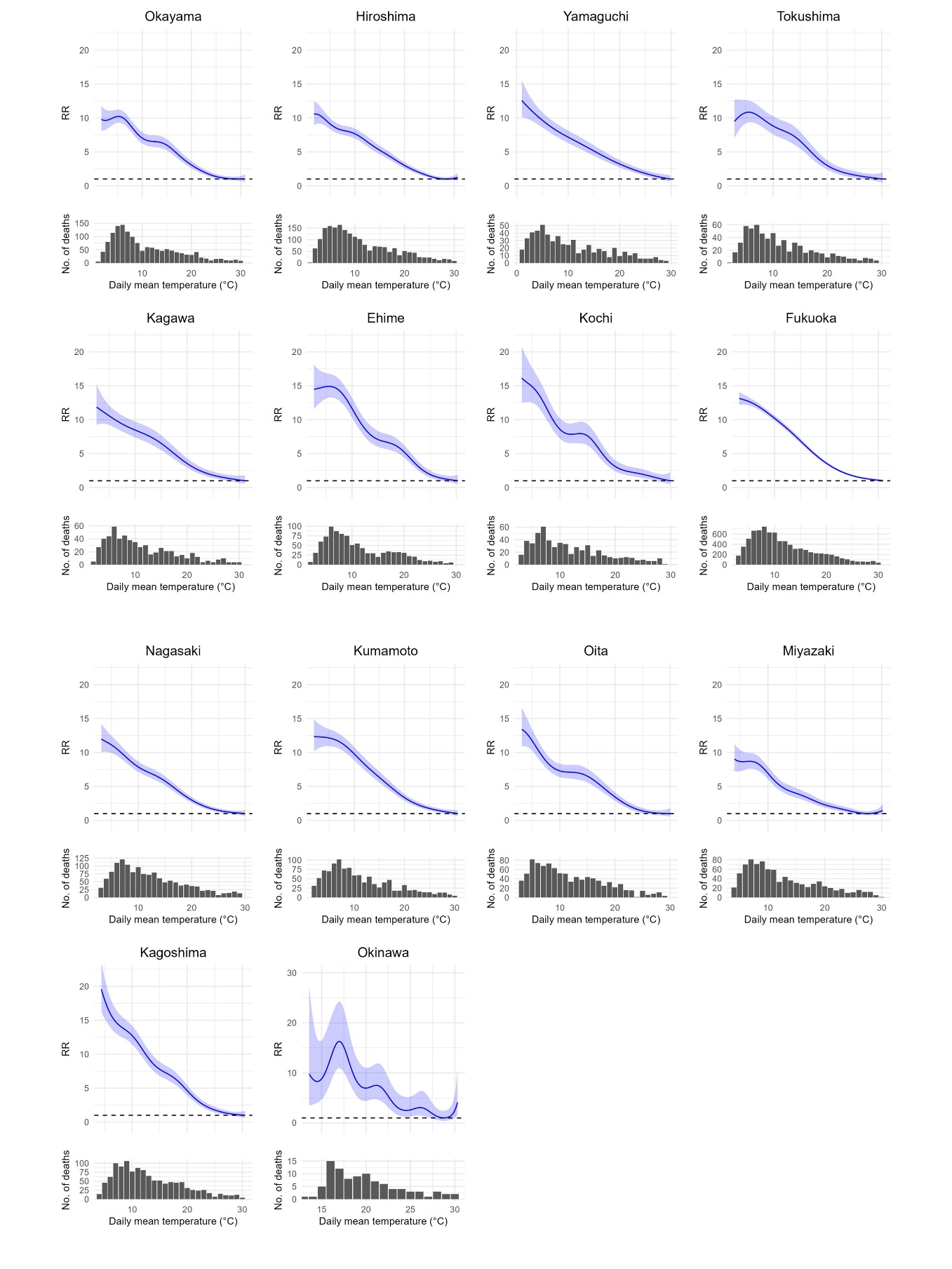
**Figure S6**. Association Between Daily Mean Temperature and Bathtub Drowning Deaths in Prefectures (Prefecture No. 33–47)

The risk of bathtub drowning deaths was estimated using a generalized additive model for each prefecture over the entire study period (1995–2020). RR values were calculated relative to the minimum predicted risk. The shaded areas indicate 95% confidence intervals. RR, relative risk.

**Figure S7.** Association Between Temperature Variables and Bathtub Drowning Deaths


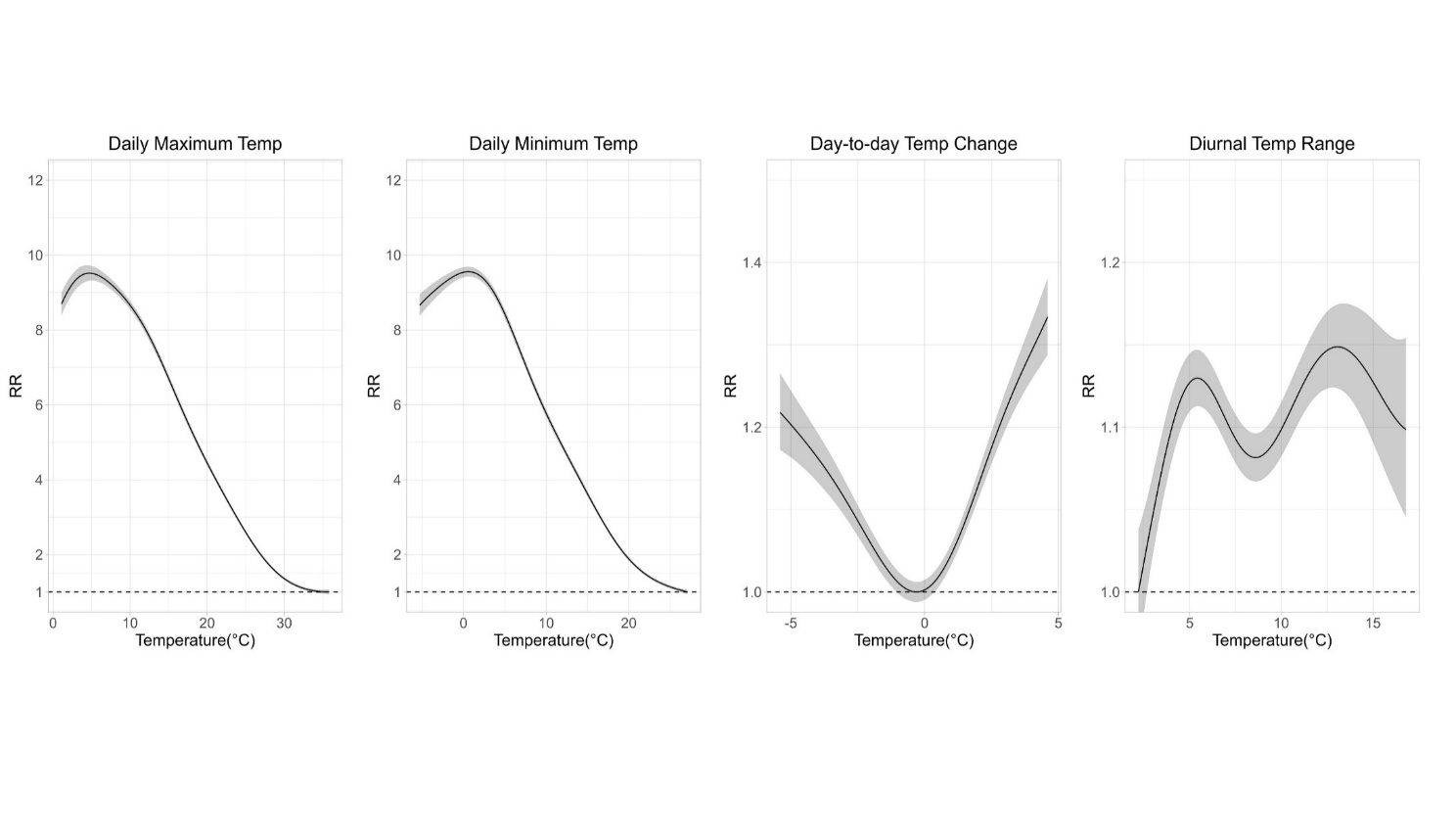


Diurnal temperature range was calculated as the difference between the maximum and minimum temperatures on days with bathtub drowning deaths. Day-to-day mean temperature change was calculated as the daily mean temperature on days with bathtub drowning deaths minus the daily mean temperature on the previous day, allowing for both positive and negative values.

The risk of bathtub drowning deaths was estimated using a generalized additive mixed model with a random intercept for each prefecture for the total population over the entire study period. RR values were calculated relative to the minimum predicted risk. The shaded areas indicate 95% confidence intervals.

RR, relative risk.

**Figure S8**. Within-Prefecture-Level Possible Modifiers of the Association Between Daily Mean Temperature and Bathtub Drowning Deaths


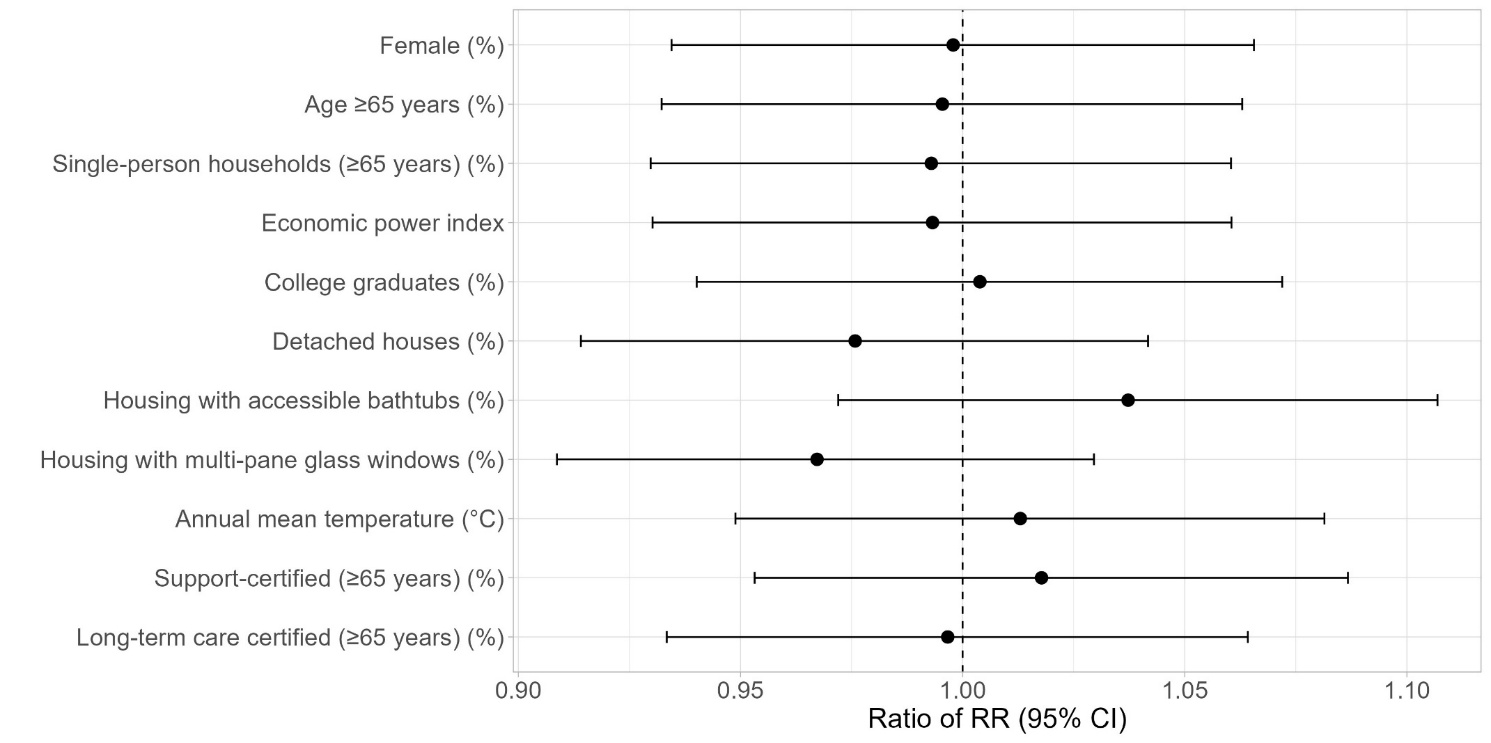


The risk of bathtub drowning deaths was estimated using a generalized additive model for each prefecture and time period (1995–2003, 2004–2012, and 2013–2020). RR values were then calculated relative to the minimum predicted risk, and the peak RR was identified. The ratio of peak RR was estimated using a meta-regression model, with the natural log-transformed peak RR as the response variable and withing-prefecture changes (one standard deviation) in prefecture characteristics as explanatory variables.

RR, relative risk.
